# Stress, drugs and the evolution of reproductive restraint in malaria parasites

**DOI:** 10.1098/rspb.2010.0564

**Published:** 2010-05-19

**Authors:** Sarah E. Reece, Eltayeb Ali, Petra Schneider, Hamza A. Babiker

**Affiliations:** 1 Centre for Immunity, Infection and Evolution, School of Biological Sciences, University of Edinburgh, Edinburgh EH9 3JT, UK; 2 Institutes of Evolution, Immunology and Infection Research, School of Biological Sciences, University of Edinburgh, Edinburgh EH9 3JT, UK; 3 Sudan Atomic Energy Commission, PO Box 3001, Khartoum, Sudan; 4 Department of Biochemistry, Faculty of Medicine, University of Khartoum, Khartoum, Sudan; 5 Biochemistry Department, Faculty of Medicine, Sultan Qaboos University, Alkhod, PO Box 35, Muscat, Oman

**Keywords:** *Plasmodium falciparum*, life-history trade-offs, gametocyte conversion, anti-malarial drug resistance, reproductive effort, resource allocation

## Abstract

Life-history theory predicts that sexually reproducing organisms have evolved to resolve resource-allocation trade-offs between growth/survival versus reproduction, and current versus future reproduction. Malaria parasites replicate asexually in their vertebrate hosts, but must reproduce sexually to infect vectors and be transmitted to new hosts. As different specialized stages are required for these functions, the division of resources between these life-history components is a fundamental evolutionary problem. Here, we test how drug-sensitive and drug-resistant isolates of the human malaria parasite *Plasmodium falciparum* resolve the trade-off between in-host replication and between-host transmission when exposed to treatment with anti-malarial drugs. Previous studies have shown that parasites increase their investment in sexual stages when exposed to stressful conditions, such as drugs. However, we demonstrate that sensitive parasites facultatively decrease their investment in sexual stages when exposed to drugs. In contrast to previous studies, we tested parasites from a region where treatment with anti-malarial drugs is common and transmission is seasonal. We hypothesize that when exposed to drugs, parasites invest in their survival and future transmission by diverting resources from reproduction to replication. Furthermore, as drug-resistant parasites did not adjust their investment when exposed to drugs, we suggest that parasites respond to changes in their proliferation (state) rather the presence of drugs.

## 1. Introduction

In its broadest sense, phenotypic plasticity is the ability of a single genotype to produce different phenotypes in different environments, through mechanisms such as differential gene expression or epigenetic effects (Schlichting & Pigliucci 1998). Phenotypic adjustment can occur in response to changes in an individual's extrinsic environment (e.g. appearance of predators) or intrinsic state (e.g. parasite burden) and is considered to be adaptive when it results in increased fitness, through enhanced survival or reproductive success (Schlichting & Pigliucci 1998). The extent to which individuals can adjust their behaviours or express different combinations of traits is constrained by resource-allocation trade-offs in which organisms have to split their finite resources between the multiple processes (e.g. growth/survival, reproduction) required to transmit gene copies to subsequent generations (Roff 1992; Stearns 1992). Life-history theory provides a solid foundation for understanding plasticity in resource-allocation trade-offs and can be used to explain and predict the evolutionary consequences of environmental variation (Roff 1992; Stearns 1992; Fischer *et al*. 2009; McNamara *et al*. 2009).

While adaptive phenotypic plasticity has been thoroughly documented for metazoan taxa (such as insects, birds and mammals), this concept has been largely overlooked for unicellular taxa such as malaria (*Plasmodium*) parasites (Reece *et al*. 2009). Malaria parasites face many analogous selective pressures and resource trade-offs to metazoans, and data suggest they also have considerable plasticity in their resource-allocation decisions (Paul *et al*. 2000; Reece *et al*. 2008, 2009). The value of an evolutionary approach for understanding how variation in the in-host environment shapes within-infection dynamics and contributes to virulence and transmission traits is increasingly being recognized. Malaria parasites replicate asexually in the circulation of their vertebrate hosts, but must produce male and female sexual stages (termed gametocytes) to transmit to vectors and subsequently infect new hosts. As in-host replication and between-host transmission are each achieved by different specialized forms, the division of resources between these life-history components is a fundamental evolutionary problem (Koella & Antia 1995). This is analogous to the problem faced by sexually reproducing organisms, in which the trade-off between investing resources into reproduction (reproductive effort) relative to growth and maintenance is a major determinant of fitness (Roff 1992; Stearns 1992).

Experiments have revealed that malaria parasites of rodents and humans increase their investment in gametocytes in response to host anaemia, changes in the age of available red blood cells (RBCs) and exposure to anti-malarial drugs (Trager & Gill 1992; Buckling *et al*. 1997, 1999*a*,*b*; Buckling & Read 1999; Trager *et al*. 1999; Reece *et al*. 2005). These observations have been interpreted as environmental changes that are stressful for parasites, to which they respond by diverting resources to gametocytes to increase between-host transmission (Taylor & Read 1997; Read & Taylor 2001). This ‘terminal investment’ is predicted by evolutionary theory when changes in intrinsic state, the environment or availability of resources reduce the probability of survival (Williams 1966). However, changes in the age of available RBCs can result in parasites having more useable resources (Paul & Brey 2003; Paul *et al*. 2003; Reece *et al*. 2005), and parasites adopt the opposite to terminal investment and are predicted to decrease investment in gametocytes in response to competitive suppression in mixed infections (Mideo & Day 2008; Pollitt *et al*. submitted). Therefore, parasites do not necessarily respond to stress by increasing investment in gametocytes, and in situations where ‘safety in numbers’ can facilitate in-host survival, ‘reproductive restraint’ is a better investment decision (Mideo & Day 2008; Reece *et al*. 2009; Pollitt *et al*. submitted). Here, we focus on testing whether parasites also adopt reproductive restraint when treated with anti-malarial drugs that suppress their proliferation.

The suggestion that malaria parasites alter investment into gametocytes when exposed to drugs has received mixed support. However, research has focused solely on whether investment is increased or not adjusted (Puta & Manyando 1997; Buckling *et al*. 1999*b*; Drakeley *et al*. 2006; Babiker *et al*. 2008; Peatey *et al*. 2009), and the alternative possibility of decreased investment (reproductive restraint) has been overlooked. Furthermore, previous studies have focused on short exposures at high doses that are insufficient to clear infections but still cause a rapid decline in parasite numbers. If rapid decline in numbers is a reliable cue of a catastrophic situation, the potential for future transmission is low, and so parasites are expected to adopt terminal investment. Here, we test whether multiple lines of the human malaria parasite *Plasmodium falciparum* adopt reproductive restraint in response to exposure to two different anti-malarial drugs that target asexual replication. We focus on providing continuous sublethal stress by treating parasites with low doses of drugs across multiple replication cycles. In endemic areas, malaria parasites face low levels of anti-malarial drugs for a variety of reasons. For example, recommended regimes can fail to eliminate parasites because treatment success is influenced by parasite density, and patients can be reinfected before previous treatments are cleared (Adjuik *et al*. 2004; Mutabingwa *et al*. 2005; Barnes *et al*. 2007). The latter is especially likely under intermittent preventive treatment, which involves giving therapeutic doses to vulnerable groups (infants and pregnant women) at regular intervals, regardless of infection status. In contrast to previous studies, we also tested parasite lines isolated from natural infections in a population where exposure to anti-malarial drugs is frequent. We used drug-sensitive and drug-resistant lines to test whether parasites alter their investment as a general response to the presence of drugs or only when vulnerable to drug treatment.

## 2. Material and methods

### (a) Parasites and cultures

The initial isolates were obtained with informed consent from patients in Asar village in Eastern Sudan. In this region, malaria transmission is markedly seasonal, following the rainy season (September to November). *Plasmodium falciparum* is the predominant parasite species, and treatment with chloroquine and/or pyrimethamine is frequent in the transmission season (Babiker *et al*. 1991). We established each isolate in *in vitro* culture according to Jensen & Trager (1977), using narrow-necked culture flasks. Briefly, each culture consisted of 5 ml complete culture medium, containing RPMI supplemented with 25 mM NaHCO3 (Sigma, UK), 25 mM 4-(2-hydroxyethyl)-l-piperazineethane-sulphonic acid (Sigma, UK), 10 per cent naive human serum, adjusted to pH 7.4, gassed with a mixture of 3 per cent O_2_, 5 per cent CO_2_ and 92 per cent N_2_, and sealed throughout incubation. We obtained distinct experimental lines by limiting dilution from isolates exhibiting diversity for genetically controlled traits including sensitivity to chloroquine and pyrimethamine (Babiker *et al*. 1991; Bayoumi *et al*. 1993). We obtained three lines sensitive to both chloroquine and pyrimethamine (identity codes: 107/89, 109/89, 104/89) and three lines resistant to both drugs (124/8, 121/89, 123/89).

### (b) Experimental procedures

We established three replicate cultures for each of our three drug-sensitive and three drug-resistant lines, and allocated one replicate per line to each of the following treatments: no-drug control, chloroquine-treated and pyrimethamine-treated. Therefore, we followed a cross-factored design in which parasites from each line experienced all experimental treatments. We set up each line/treatment combination in duplicate or triplicate, resulting in six to nine cultures for each of our six lines, totalling 39 cultures. We followed Carter *et al*. (1993) to establish gametocyte-producing cultures: as soon as 5 per cent of RBCs were parasitized, 1 ml (5%) was added to 4 ml of 6 per cent haematocrit-uninfected blood, in media appropriate to the treatment group for each culture (media without drugs, with 0.5 pmol chloroquine or with 0.5 nM pyrimethamine) and the haematocrits of all cultures were set to 6 per cent (no further RBCs were added during culturing). The doses of chloroquine and pyrimethamine we used are low: the minimum doses required to clear all sensitive parasites are 8 pmol and 10^−5^ M, respectively. We maintained cultures for 11 days and replaced culture medium daily with either drug-free or drug-containing media according to the treatment for each culture.

We sampled cultures daily using Giemsa-stained thin blood smears and recorded the parasitaemia, the numbers of ring stages and stage 2 gametocytes per 10 000 RBCs. We used these values to calculate the gametocyte conversion rates for each culture from day 0 to day 8, following Carter & Miller (1979). The gametocyte conversion rate (reproductive effort) represents the proportion of a cohort of asexually produced ring-stage parasites that become sexual-stage gametocytes. After invading an RBC, parasites spend their first 24 h as ring stages, which can be readily distinguished in blood smears. Gametocytes cannot reliably be distinguished by morphology until they have reached stage 2 of their development, 48 h after invasion. Therefore, the conversion rate on day *t* is simply calculated as the number of stage 2 gametocytes observed in 10 000 RBCs on day *t* + 2 divided by the number of ring-stage asexual parasites observed in 10 000 RBCs on day *t*. Unlike *in vivo* infections, *in vitro* culture is ideal for calculating conversion rates because all parasites of all developmental stages are accessible for sampling (gametocytes cannot sequester in tissues and asexuals are not exposed to attack from immune factors), so potential problems such as stage-specific mortality cannot confound estimates.

### (c) Statistical analysis

We used R v. 2.5.0 (The R Foundation for Statistical Computing, Vienna, Austria) for all analyses. We log-transformed conversion rates to conform to the assumptions of parametric tests and used linear mixed-effects models with maximum likelihood to investigate the effects of drug treatment and drug sensitivity on the patterns of conversion rates observed during cultures, according to the three sets of analysis described below. Mixed-effects models allowed each culture to be nested within the identity of the line contributing the parasites while fitting line identity as a random effect, which can account for problems of non-independence associated with repeated measures in longitudinal analyses. We nested culture within the identity of each line for all analyses and evaluated the significance of fixed effects by comparing models using log-likelihood ratio tests following stepwise deletion of the least significant term. Specifically, we compared the change in model deviance, following term deletion, to *χ*^2^ distributions with degrees of freedom corresponding to the difference in the number of terms in the models. We simplified maximal models until only significant terms remained in the model (Pinheiro & Bates 2000). Minimal models from our different analyses were compared with log-likelihood ratio tests and Akaike information criteria (AIC). We then re-ran our final minimal model using restricted maximum likelihood to estimate the effect sizes and plot model predictions.

We ran three sets of models to find the most parsimonious description for the influences of drug treatment and susceptibility to drugs on the patterns of gametocyte conversion rates observed during cultures. By comparing models in which we combined different factor levels and different treatment groups, we tested whether gametocyte conversion rates differed between cultures according to the type of drug used and the susceptibility of parasites. In addition to testing for the effects of drug treatment or drug sensitivity, we also fitted RBC density as a covariate in all maximal models as host anaemia has been shown to influence conversion rate during *in vivo* infections (Reece *et al*. 2005). In all analyses, day was fitted as a covariate. To test whether patterns of conversion were nonlinear, each maximal model contained both linear and quadratic terms for day, but, in all models, all interactions and main effects for the quadratic were non-significant (*p* > 0.05).

In our first analysis, we specified drug treatment as a fixed factor with three levels (chloroquine, pyrimethamine, control) and we specified whether parasites were resistant or sensitive as a fixed factor with two levels. Second, we tested whether the type of drug mattered by combining the chloroquine and pyrimethamine cultures and fitting drug treatment as a fixed factor with two levels (drugs, control). Third, we investigated whether our analysis could be simplified further by testing whether gametocyte conversion rates only differed between sensitive parasites in drug-treated cultures and all other groups that were either in control cultures or resistant to drugs. We fitted this as a fixed factor with two levels, by classifying parasites as safe (i.e. combining control cultures and resistant lines in drug-treated cultures) or vulnerable to drugs in their cultures (i.e. only sensitive lines in drug-treated cultures). By comparing the resulting minimal models from these three analyses, we obtained the most parsimonious description of conversion rates without significant loss of explanatory power. Finally, we tested whether the fixed effects providing the best description of conversion rates also influenced the densities of gametocytes and asexual stages observed during days 1–8 in the cultures.

## 3. Results

Our first analysis revealed that the patterns of conversion rates observed during cultures were significantly influenced by the interaction between treatment, the drug sensitivity of parasites and day (*χ*^2^_2_ = 6.49; *p* = 0.039), but not by RBC density (*χ*^2^_1_ = 0.96; *p* = 0.328). Our second analysis, in which we collapsed the chloroquine- and pyrimethamine-treated groups into one factor level (table 1), also revealed that patterns of conversion rates were significantly influenced by the interaction between treatment and whether parasites were sensitive or resistant (*χ*^2^_1_ = 5.81; *p* = 0.016), but not by RBC density (*χ*^2^_1_ = 0.82; *p* = 0.365). We compared the minimal models for our first and second analyses (AIC = 890.49 and 887.81, respectively), and found that there was no significant loss of deviance associated with the second, simpler model (*χ*^2^_4_ = 5.32; *p* = 0.256), revealing that patterns of conversion rates did not significantly differ according to the type of drug parasites were treated with and that resistant parasites do not alter their conversion rates in response to drugs.

**Table 1.** Model estimates (±s.e.) and significance for the intercepts and slopes for transformed conversion rates examined in our second analysis, revealing that the conversion rates of sensitive (vulnerable) parasites exposed to drugs are significantly lower than for other treatment groups (safe from drugs), and that conversion rates of parasites safe from drugs were not significantly different from each other. Estimates (±s.e.) given are relative to the conversion rates of the resistant parasites in control cultures, and *t-* and *p*-values test the difference between the resistant parasites in control cultures and the other groups.

		*t*	*p*
*intercepts*			
resistant parasites without drugs	−3.545 (±0.25)		
Δ resistant parasites exposed to drugs	0.166 (±0.30)	0.56	0.578
Δ sensitive parasites without drugs	0.242 (±0.37)	0.66	0.543
Δ sensitive parasites exposed to drugs	0.400 (±0.44)	0.91	0.370
*slopes*			
resistant parasites without drugs	0.278 (±0.04)		
Δ resistant parasites exposed to drugs	−0.007 (±0.05)	0.15	0.879
Δ sensitive parasites without drugs	−0.007 (±0.06)	0.12	0.901
Δ sensitive parasites exposed to drugs	− 0.167 (±0.07)	2.41	0.017

**Figure 1.**
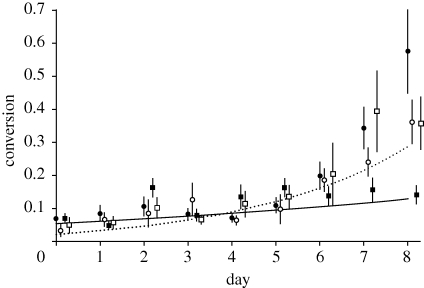
Means ± s.e. gametocyte conversion rates (calculated as the proportion of ring stages from each asexually produced cohort that committed to becoming gametocytes) observed during cultures for drug-sensitive and drug-resistant isolates when exposed to anti-malarial drugs or control conditions. Conversion rate significantly differed according to whether parasites were ‘safe’ or ‘vulnerable’ to any drugs in their cultures. Parasites safe from treatment included those in control cultures (white squares, sensitive lines; white circles, resistant lines) and resistant parasites in drug-treated cultures (black circle), whereas vulnerable parasites are the drug-sensitive parasites exposed to drugs (black squares). Therefore, only the sensitive parasites altered their conversion rates when exposed to drugs. Best-fit lines are from the minimum adequate model for parasites vulnerable to drugs (solid line) and safe from drugs (dashed line).

We then tested whether gametocyte conversion rates simply depended upon whether parasites were both in drug-treated cultures and susceptible to drugs. This analysis revealed that parasites vulnerable to drugs in their cultures (the sensitive lines in drug-treated cultures) followed patterns with significantly lower conversion rates than parasites safe from drugs (figure 1; *χ*^2^_1_ = 23.74; *p* < 0.0001). There was no additional influence of whether parasites were drug-sensitive or resistant (*χ*^2^_1_ = 0.34; *p* = 0.557; interaction with day: *χ*^2^_1_ = 0.81; *p* = 0.367), nor of RBC density (*χ*^2^_1_ = 0.002; *p* = 0.969). Again, there was no significant loss of deviance when comparing the minimal models for our second and third analyses (*χ*^2^_6_ = 0.76; *p* = 0.944; AIC = 887.81 and 880.57, respectively). This shows that conversion rates simply depend upon whether parasites were vulnerable to, or safe from, drugs they were exposed to in their cultures.

**Figure 2.**
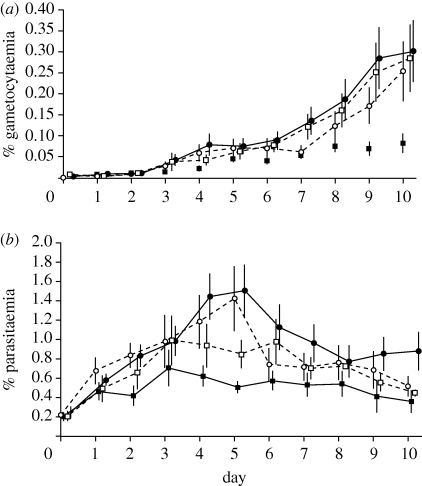
(*a*) Means ± s.e. per cent of RBCs infected with gametocytes (gametocytaemia) and (*b*) ring-stage asexually produced parasites (parasitaemia) during cultures for drug-sensitive (squares) and drug-resistant (circles) lines when exposed to anti-malarial drugs (black symbols) or control conditions (white symbols).

In addition, we investigated whether the densities of gametocytes and asexual stages also differed according to whether parasites were vulnerable to, or safe from, drugs in their cultures. As expected, the lower conversion rates observed when parasites were vulnerable to drugs translated into the production of fewer gametocytes compared with parasites safe from drugs (figure 2*a*; *χ*^2^_8_ = 36.20; *p* < 0.0001). In contrast, while the densities of asexual parasites varied during cultures (*χ*^2^_8_ = 164.11; *p* < 0.0001), this was not significantly influenced by whether parasites were vulnerable to drugs (figure 2*b*; *χ*^2^_1_ = 1.47; *p* = 0.225; interaction with day: *χ*^2^_8_ = 8.63; *p* = 0.374). Moreover, the asexual densities of the sensitive lines did not differ significantly between treatments (*χ*^2^_1_ = 1.65; *p* = 0.199).

## 4. Discussion

We tested whether multiple lines of the human malaria parasite (*P. falciparum*) facultatively alter their allocation of resources to between-host transmission (gametocyte conversion rate) when exposed to low doses of different anti-malarial drugs. Our analyses show that gametocyte conversion rates were significantly lower in the drug-sensitive lines when exposed to drugs than for all parasites lines in drug-free control cultures, and than for the drug-resistant lines in drug-treated cultures (figure 1). Put simply, only the drug-sensitive lines decreased their investment into transmission stages when exposed to drugs. These data reveal that, as predicted, parasites adopt reproductive restraint when stressed by low doses of drugs. The drug-resistant lines did not significantly alter their conversion rates when exposed to drugs (and their conversion rates were not significantly different from the sensitive lines in control cultures), suggesting that parasites do not respond directly to drugs but instead adjust their conversion rates in response to changes in their proliferation (Koella & Antia 1995; Paul & Brey 2003). Information about their absolute density, or changes in density, could be obtained through a form of quorum sensing (Dyer & Day 1993) or the presence of lysed parasites, and other studies also suggest that parasites respond to their own density (Reece *et al*. 2008; Pollitt *et al*. submitted). Alternatively, as our lines were isolated at a time of frequent exposure to drugs, it is possible that the sensitive lines have evolved a specific response to these drugs, which the resistant parasites either never had or have subsequently lost. The latter could occur if there are costs associated with maintaining unnecessary information-gathering and processing systems (DeWitt *et al*. 1998). Investigating how parasites respond to drugs with different modes of action and different target stages could resolve this.

Our data reveal that parasites adopt reproductive restraint when exposed to low doses of anti-malarial drugs. This response is consistent with the hypothesis that reducing investment in gametocytes enables parasites to prioritize their survival by increasing their replication rate (Roff 1992; Stearns 1992; Koella & Antia 1995; Mideo & Day 2008; Fischer *et al*. 2009; McNamara *et al*. 2009). The asexual densities of sensitive strains were not significantly reduced in the drug-treated cultures, and we suggest that reproductive restraint enabled these parasites to reduce the detrimental effect of drugs. Quantifying the survival advantage gained by reducing investment in gametocytes requires experiments that measure the fitness consequences for parasites unable to respond to drugs, which in turn requires indentifying the mechanism parasites use to measure and respond to stress. This is also an area where mathematical modelling would be very useful. By calculating how much replication can be increased for a given reduction in gametocyte investment, the optimal strategy for parasites experiencing different probabilities of survival can be predicted. This would reveal whether parasites should switch from reproductive restraint to terminal investment, and where this threshold lies. More broadly, combining experiments and models in this way could provide novel insight into the costs and limits of phenotypic plasticity (Auld *et al*. 2009; McNamara & Houston 2009). Alternatively, it may be the case that our low drug doses were not harmful to the sensitive strains. However, this explanation is not as parsimonious, because it is not clear why the sensitive parasites would respond to drugs that are not harmful, and suggests that sensitive parasites respond to the presence of drugs, in which case it is not clear why the resistant parasites do not respond to drugs too.

Previous studies have revealed that malaria parasites of rodents and humans increase their investment in gametocytes when exposed to doses of drugs. Why have we recovered the opposite result? In addition to previous studies using higher doses and applying them for a short time, we outline three other explanations, which may not be mutually exclusive, below. First, our *in vitro* approach and analysis of patterns of conversion differ from previous studies. Parasite behaviour varies considerably during *in vivo* infections, and even with seemingly simple experimental manipulations, variation in potentially confounding factors (e.g. changes in RBC age distribution, anti-gametocyte immunity) can also occur (Reece *et al*. 2005, 2009; Long *et al*. 2008; Mideo *et al*. 2008). In natural infections, drug treatment often coincides with, and can alter, the development of disease symptoms, including anaemia (Ekvall *et al*. 1998), which could confound apparent responses to drug treatment. While the influences of these factors could be tested, and potentially controlled for, they are rarely measured as analyses are usually focused on summary or snapshot data from infections.

Second, our results may differ from experiments with rodent parasites (Buckling *et al*. 1997; Buckling & Read 1999) because the developmental schedules of rodent and human parasites differ. The ability to match investment in gametocytes to in-host conditions may be constrained by how much the in-host environment can change during a cell cycle and during the maturation period of gametocytes. The cell cycle of rodent parasites is 24 h and gametocytes require less than 48 h to reach maturity, whereas *P. falciparum* parasites only replicate every 48 h and gametocytes require around 10 days to reach maturity (Garnham 1966). Rodent malaria parasites may be able to rapidly switch strategies, but *P. falciparum* parasites might be constrained to playing it safe and investing more in survival when in-host conditions are changing.

Third, our results may differ from previous studies with *P. falciparum* owing to variation in the ecology of the populations that isolates were collected from. Previous studies have used *P. falciparum* parasites from regions of high prevalence and year-round transmission, which are also well adapted to *in vitro* growth (Buckling *et al*. 1999*b*). Exposure to drugs may be a selection pressure that is too novel for these parasites to respond optimally to, and/or a fast drop in density may be used as a cue that survival is unlikely. In contrast, our lines have been maintained in an *in vitro* culture for only a limited time, and were isolated from an area with low and seasonal transmission, in which chronic infections are responsible for much of the malaria transmission and anti-malarial drug use is common (Babiker *et al*. 1991, 1998). Parasites in areas of high transmission may benefit from a ‘live fast, die young’ life history (Lines *et al*. 1991), but the value of future versus current reproduction is likely to be much higher for our parasites, and so they should be more likely to invest in survival when stressed.

Observations of changes in gametocyte carriage following drug treatment of natural infections in eastern Sudan—the region from which our parasites were isolated—are consistent with our results (Nassir *et al*. 2005; Ali *et al*. 2006). This issue is controversial because is not clear whether elevated gametocyte densities observed in natural infections after drug treatment are due to a change in investment or simply due to the release of sequestered gametocytes (Drakeley *et al*. 2006; Babiker *et al*. 2008). Our data suggest the latter, and if this occurs in natural infections, there will be implications for understanding and predicting the spread of drug resistance (Read & Huijben 2009). By reducing conversion rates in response to drug treatment, drug-sensitive parasites may transmit at a slower rate than drug-resistant parasites during exposure, but will be more likely to survive and, thus, transmit over a longer duration than currently expected. Determining whether the response of sensitive parasites to drugs is a general response to stresses that influence their proliferation or a specific adaptation to drugs now is central to predicting the behaviour and evolutionary trajectories of parasites in populations with different exposure to drugs and transmission patterns. The opportunity to combine experiments with field data for the same population of human parasites offers a promising way to further the field of evolutionary medicine (Williams & Nesse 1991; Stearns & Koella 2007). Mathematical models, experiments and field data are now required to determine how parasite investment strategies influence the maintenance of drug-sensitive parasites in natural populations, how their investment strategies vary with the magnitude and form of the stress they experience, and what the implications are for their virulence (Schneider *et al*. 2008). More generally, using an evolutionary approach to explain virulence hinges on the useful application of life-history trade-offs to parasites (Day 2003; Alizon *et al*. 2009). By revealing that malaria parasites trade off survival and reproduction in the manner explained by evolutionary theory for life histories, we can be more optimistic about understanding virulence.
